# Phytochemical Diversity of *Punica granatum* L. and Its Multi-Target Biological Functions

**DOI:** 10.3390/nu18081306

**Published:** 2026-04-21

**Authors:** Zofia Kobylińska, Aleksandra Bochno, Ewelina Och, Martyna Kotula, Patrycja Kielar, Sabina Galiniak, Mateusz Mołoń

**Affiliations:** 1 Department of Genetics, Faculty of Biology, Natural Protection, and Sustainable Development, University of Rzeszow, al. Tadeusza Rejtana 16C, 35-959 Rzeszów, Poland; zkobylinska@ur.edu.pl (Z.K.); ab125029@stud.ur.edu.pl (A.B.); eo122284@stud.ur.edu.pl (E.O.); mk125611@stud.ur.edu.pl (M.K.); 2 Department of Medical Chemistry and Metabolomics, Faculty of Medicine, University of Rzeszów, al. Tadeusza Rejtana 16C, 35-959 Rzeszów, Poland; pkielar@ur.edu.pl

**Keywords:** *Punica granatum*, polyphenols, punicalagin, inflammation, microbiota, pharmacology

## Abstract

*Punica granatum* L. is a nutritionally relevant fruit with a complex phytochemical profile that varies across its anatomical fractions, including peel, arils, juice, seeds, and seed oil. Although pomegranate is widely recognized for its health-promoting potential, the nutritional significance of its matrix-dependent composition, bioavailability, and gut microbiota-mediated metabolism remains insufficiently integrated. This review aimed to critically evaluate the phytochemical diversity of pomegranate and its nutrition-related multi-target biological functions, with particular emphasis on food matrices, bioaccessibility, and translational relevance. A structured review of peer-reviewed studies indexed in major scientific databases from 2000 to January 2026 was conducted. Eligible reports included analytical, preclinical, and clinical studies addressing the composition of pomegranate-derived materials and their biological effects, with attention to extraction matrix, processing, bioavailability, microbial biotransformation, and mechanisms of action. Pomegranate exhibits marked matrix-specific phytochemical diversity. Peel is particularly rich in ellagitannins, especially punicalagin and punicalin; arils and juices are enriched in anthocyanins and flavonols; and seed oil contains high levels of punicic acid. Reported biological activities include antioxidant, anti-inflammatory, antimicrobial, metabolic, anti-aging, and anticancer effects. These actions appear to result from synergistic interactions among multiple bioactive compounds rather than from a single dominant constituent. Importantly, gut microbiota-driven conversion of ellagitannins and ellagic acid into urolithins is a major determinant of systemic bioactivity and may contribute to interindividual variability in response. The health effects of pomegranate should be interpreted within a nutrition-focused, matrix-dependent framework integrating composition, processing, bioavailability, and microbiota-derived metabolism.

## 1. Introduction

The pomegranate (*Punica granatum* L.), commonly referred to as the pomegranate fruit, is regarded as one of the oldest and most symbolically significant cultivated plant species in human history. It is considered among the earliest edible plants subjected to domestication, alongside the fig tree, date palm, grapevine, and olive tree [1]. Throughout history, the pomegranate has played an important role in the culture and symbolism of numerous civilizations. It appears in religious and mythological narratives, including the Bible, the Qur’an, Greek and Turkish mythology, as well as in Buddhist tradition and Chinese art [2,3].

Its Latin name derives from the words *pomum* (apple) and *granatus* (grainy or seeded), reflecting historical nomenclature in which pomegranate fruits were described as “seeded apples” [2]. Since ancient times, the exceptional medicinal properties of the pomegranate have been recognized, leading to its association with symbolism related to life, fertility, growth, immortality, and prosperity [1,4].

The pomegranate (*Punica granatum* L.) is an ancient fruit species with a broad geographic distribution, shaped by its high adaptive capacity and long history of cultivation [5,6]. The species originated in the Middle East, most likely in the region of present-day Iran, from where it gradually spread to ancient Egypt, Greece, and Italy, and subsequently to the Iberian Peninsula, encompassing modern-day Spain and Portugal [3,7,8].

Archaeobotanical evidence indicates that the pomegranate was domesticated at a very early stage, dating back to the Neolithic period. Well-preserved remains of this plant have been identified from the Bronze Age, particularly in the Levant region (including the areas of Jericho and Arad). Even more numerous finds from the Late Bronze Age attest to the significant role of pomegranate in Eastern Mediterranean trade; fruits of this species were discovered, among others, in the cargo of the Uluburun shipwreck [1,2,9].

The pomegranate was most likely introduced into Asia via the Silk Road, reaching China and Japan through India. During the Age of Great Geographical Discoveries, Spanish explorers contributed to the introduction of pomegranate into North and Central America. At present, its cultivation extends across numerous tropical and subtropical regions worldwide [1,4].

Existing pomegranate reviews commonly summarize the presence of major polyphenol classes and catalog reported biological activities; however, they often treat phytochemistry and bioactivity as separate narratives and give limited attention to the food-system and clinical determinants of efficacy, including processing-related transformations, matrix-dependent release during digestion, lack of harmonized compositional reporting across products, and major sources of between-study heterogeneity such as formulation type, phytochemical standardization, dose, intervention duration, and study population characteristics. Moreover, mechanistic claims are frequently extrapolated from preclinical models without sufficient discussion of bioavailability constraints and the metabolite-centric nature of pomegranate effects in humans, particularly the pivotal role of gut microbiota in generating urolithins and driving responder–nonresponder variability. To address these gaps, this review consolidates evidence into a translational chain that links product/matrix characteristics to phytochemical profiles, bioaccessibility and metabolism, and downstream multi-target biological outcomes. We emphasize critical interpretation of discrepant findings, propose practical standardization markers for improved comparability, and outline research priorities needed to move from promising mechanistic data toward reproducible, clinically relevant evidence.

## 2. Review Methodology

This review was conducted as a structured narrative review with a primary focus on the chemical composition, analytical characterization, and food-relevant bioactivity of *Punica granatum* L. Literature searches were performed using the Web of Science Core Collection, Scopus, PubMed, and ScienceDirect databases. Peer-reviewed articles published between January 2000 and January 2026 were considered, with emphasis on studies reflecting recent advances in food analytical chemistry and phytochemical profiling. Search queries combined the terms “*Punica granatum*”, “pomegranate”, “polyphenols”, “ellagitannins”, “punicalagin”, “ellagic acid”, “anthocyanins”, “pomegranate peel”, “pomegranate seeds”, “pomegranate seed oil”, “fatty acids”, “phytochemical composition”, “antioxidant activity”, “food chemistry”, “functional food”, and “gut microbiota”. Additional relevant publications were identified by screening reference lists of selected articles. Studies were included if they reported chemical characterization of pomegranate-derived materials (fruit, peel, seeds, juice, or extracts) using analytical techniques, including HPLC (High-Performance Liquid Chromatography), UHPLC–MS (Ultra-High-Performance Liquid Chromatography coupled with Mass Spectrometry), GC–MS (Gas Chromatography–Mass Spectrometry), and NMR (Nuclear Magnetic Resonance spectroscopy). or validated spectrophotometric assays. Articles evaluating bioactive properties relevant to dietary intake, particularly antioxidant, antimicrobial, and metabolic effects, were considered when the tested materials were clearly defined and chemically characterized. Studies addressing processing effects, bioaccessibility, biotransformation, and structure–activity relationships of pomegranate constituents were also included. From each eligible study, data were extracted on the plant part analyzed, processing or extraction conditions, analytical methodology, qualitative and quantitative phytochemical profiles, and reported biological effects. In addition to compositional and mechanistic data, we extracted information on major sources of heterogeneity across studies, including formulation type, plant fraction, extraction and processing conditions, marker-compound standardization, daily dose, intervention duration, and population characteristics in human studies. These variables were considered in the cross-study interpretation of reported biological effects. Particular attention was given to major compound classes, including ellagitannins (notably punicalagin), ellagic acid and its metabolites, flavonoids, anthocyanins, and seed-derived fatty acids such as punicic acid. Where feasible, quantitative data were compared across studies in a structured manner; however, complete harmonization of reported units was not always possible because of differences in matrices, analytical methods, extraction procedures, and data normalization approaches.

This review was designed as a structured narrative review intended to integrate phytochemical, mechanistic, preclinical, and clinical evidence relevant to pomegranate-derived products. Therefore, formal systematic review procedures, including a PRISMA flow diagram and study quality or risk-of-bias assessment, were not applied.

## 3. Biology of *P. granatum*

From a taxonomic perspective, the genus *Punica* comprises two species: *P. granatum*, the cultivated species, and *P. protopunica*, which is endemic to the island of Socotra and is often regarded as an ancestral form of the cultivated pomegranate. Historically, the genus *Punica* was classified within the family Punicaceae [2,10].

*Punica granatum* is a deciduous shrub with an irregular growth habit, typically reaching a height of 1.5–7 m [6]. The pomegranate fruit represents a specific type of berry known as a balausta, i.e., a many-seeded fruit with a leathery pericarp derived from an enlarged ovary and persistent perianth. The mature fruit is spherical, with a diameter of 5–12 cm, and is covered by a hard, leathery rind ranging in color from yellowish-brown to deep purplish red. The interior of the fruit is divided by white, spongy septa into numerous chambers containing juicy, ruby-colored arils that surround the seeds. These arils constitute the edible portion of the fruit. Each aril encloses a single seed, which is typically hard and angular [11,12]. The pomegranate thrives particularly well under dry and semi-arid climatic conditions, characterized by low annual precipitation and limited water availability. Its ability to grow on a wide range of soil types and under diverse climatic conditions has contributed to its broad geographical distribution [8,13]. In warm climates, plants may exhibit evergreen behavior [6].

Within the species, numerous ornamental forms are also present, including dwarf varieties and cultivars with double flowers, some of which are functionally sterile. Chromosome numbers vary among cultivars and typically equal 2*n* = 16; however, other chromosome numbers and polyploid lines have also been reported [6].

## 4. Chemical Composition

### 4.1. General Phytochemical Overview

*P. granatum* is characterized by a rich content of biologically active chemical compounds. These include polyphenols, flavonoids, anthocyanins, sterols, alkaloids, tannins, and terpenes, as well as fatty acids, lignins, saccharides, and vitamin C [14]. The concentration of individual compounds varies depending on the cultivar, harvest time, and cultivation conditions [15,16]. In a study conducted on 22 pomegranate cultivars, the total polyphenol content in juice ranged from 0.81 to 1.52 mg gallic acid equivalents (GAE)/mL. Significant differences among cultivars were also observed in the total antioxidant capacity of pomegranate juice, which ranged from 3.44 to 6.81 mg Trolox equivalents (TE)/mL [16]. Antioxidant levels are influenced by factors such as light exposure, soil chemical composition, air humidity, temperature, atmospheric pressure, and other environmental parameters [15]. Distinct parts of the plant exhibit different chemical profiles. According to a study by Marsoul et al., extracts from the pomegranate trunk are dominated by phenolic compounds and flavonoids, with a total phenolic content of 272.82 ± 32.05 µg/mL of extract and flavonoid content of 387.25 ± 1.75 µg/mL of extract. The trunk also contains sterols and polyterpenes in smaller amounts [17]. The fruit peel is particularly rich in tannins, flavonoids, alkaloids, quinones, terpenoids, phenolic compounds, coumarins, and steroids [18]. It is also a substantial source of dietary fiber. It has been demonstrated that administration of powdered pomegranate peel as a fiber source to hypercholesterolemic rats reduces body weight gain and limits lipid peroxidation [19].

### 4.2. Polyphenols

Eighteen phenolic compounds have been identified in pomegranate peel, of which sixteen belong to the tannin group. Punicalagin, together with its isomers, as well as gallic acid and ellagic acid, constitutes the most abundant group of polyphenols in pomegranate [20,21]. Punicalagin inhibits the production of pro-inflammatory cytokines in macrophages and also prevents autophagy [22]. Moreover, it exhibits neuroprotective effects, which may contribute to the prevention of neurodegenerative diseases [23]. Its bactericidal and bacteriostatic activity against multidrug-resistant bacterial strains belonging to the family *Enterobacteriaceae* has also been reported [24]. Punicalagin and ellagic acid display antidepressant and anxiolytic properties and reduce the levels of reactive oxygen species (ROS) in the brains of rats exposed to FeSO_4_ and peroxynitrite [25]. Other tannins present in pomegranate fruit include castalagin, casuarinin, epicatechin, and gallotannins [14].

Phenolic acids identified in *P. granatum* include gallic, caffeic, ferulic, and cinnamic acids, with their concentrations being influenced by environmental conditions during cultivation. Pomegranate peel contains 123.79 mg of gallic acid per 100 g, while caffeic acid accounts for 20.56 mg/100 g of dry matter [26].

The total polyphenol content in juice obtained from the whole fruit (including the peel) is approximately 2566 mg/L and is significantly higher than in juice prepared solely from pomegranate arils, in which total polyphenol levels range from 1800 to 2100 mg/L [27]. The total polyphenol content decreases as the fruit ripens [28].

### 4.3. Flavonoids

Fifty flavonoids have been identified in pomegranate peel, including 6 flavan-3-ols, 13 flavonols, 4 flavanones, 1 dihydrochalcone, 3 isoflavones, 6 flavones, 2 flavanonols, 9 anthocyanins, 3 procyanidins, 2 aurones, and 1 chromone [21]. Flavonoids present in the peel include, among others, luteolin, quercetin, gallocatechin, epicatechin, and kaempferol [20].

Among the flavonoids found in pomegranate fruits, anthocyanins, catechins (flavanols), and flavonols are of particular importance [29]. Anthocyanins are mainly present in the arils and are responsible for the characteristic red coloration of the fruit. The most important pomegranate anthocyanins include pelargonidin-3-glucoside, cyanidin-3-glucoside, delphinidin-3-glucoside, cyanidin-3,5-diglucoside, and delphinidin-3,5-diglucoside [30]. Delphinidins present in pomegranate have been shown to inhibit the proliferation of breast cancer cells [31].

### 4.4. Fatty Acids and Organic Acids

The total fat content in pomegranate varies depending on the cultivar and cultivation site and ranges from 4.44% to 24% [32,33]. Pomegranate seed oil contains punicic acid, which constitutes approximately 76% of all fatty acids in the seeds, as well as linoleic acid, oleic acid, and 42 other fatty acids [34,35]. Punicic acid is a polyunsaturated fatty acid and an isomer of linoleic acid, with pomegranate seeds being its primary natural source. It exhibits particularly valuable antioxidant, anticancer, and anti-inflammatory properties [36] and has also been shown to prevent the development of diabetes and obesity [37].

### 4.5. Tissue-Specific Bioactive Composition of P. granatum L.

Individual anatomical fractions of pomegranate are characterized by clearly distinct phytochemical profiles, reflecting differences in compound polarity, intestinal availability, and downstream metabolic processing. As a result, the biological properties attributed to pomegranate are strongly dependent on the type of preparation used—such as aril-derived juice, peel extracts, or seed oil—and should not be generalized across products in the absence of compositional comparability and rigorous chemical profiling. Current syntheses of the literature consistently describe pomegranate as a multi-constituent system acting on multiple biological targets, rather than as a source of a single dominant active compound (Table 1). This interpretation is in line with broader literature on fruit phytochemistry emphasizing that biologically active fruit matrices should be viewed as complex systems of interacting compounds rather than as sources of single isolated actives [38]. Observed effects are typically linked to the combined modulation of oxidative balance and inflammatory signaling, while systemic outcomes in vivo are further shaped by gut microbiota-mediated transformation of ellagitannins and ellagic acid into urolithins, which may account for a substantial proportion of downstream bioactivity [14,29,39]. From a translational perspective, the practical relevance of pomegranate-derived products depends not only on the presence of bioactive compounds, but also on the specific formulation used, the plant fraction selected, and the degree of phytochemical standardization. Peel extracts, juices, and seed oil represent chemically distinct preparations with different dominant constituents, bioavailability profiles, and potential fields of application. Therefore, the interpretation of biological effects in nutritional and nutraceutical contexts should take into account processing conditions, extraction protocols, and the use of chemically defined marker compounds to improve product comparability and reproducibility.

#### 4.5.1. Peel

Among the anatomical fractions of pomegranate, the peel is consistently identified as the richest source of hydrolyzable tannins, with ellagitannins—most notably punicalagin and punicalin—occurring at markedly higher concentrations than in other tissues, together with a range of additional phenolic compounds [40,41]. From a pharmacological standpoint, this enrichment is relevant because ellagitannins combine pronounced redox activity with strong affinity for proteins, a property that underlies their broad bioactivity but also renders their effects highly dependent on the extraction matrix and processing conditions. Comparative extraction studies have shown that the choice of solvent exerts a decisive influence on both the qualitative composition and quantitative abundance of peel polyphenols, leading to substantial differences in measured antioxidant, antibacterial, and anti-inflammatory activities. Such methodological variability represents a major contributor to between-study heterogeneity and provides a strong rationale for standardization strategies based on chemically defined marker compounds—such as punicalagin—alongside transparent reporting of extraction protocols [20,41]. At the mechanistic level, ellagitannin-rich peel fractions are repeatedly linked to attenuation of pro-inflammatory signaling and to antimicrobial activity in vitro. Bioassay-guided investigations have further identified punicalagin as a principal contributor to activity against *S. aureus*, supporting its dual role as a functional driver and a practical quality-control marker in peel-derived formulations designed for antimicrobial applications [42]. In addition to antibacterial effects, hydrolyzable tannins isolated from pomegranate have demonstrated antifungal activity, including inhibition of fungal DNA topoisomerases, indicating that tannin-dominated fractions can exert broader anti-infective actions [43]. Evidence for antiviral activity has also been reported for peel polyphenols, with experimental studies describing interference at early stages of host–virus interactions, such as reduced binding between the SARS-CoV-2 spike protein and the ACE2 receptor in vitro. These observations should, however, be viewed as indicators of mechanistic plausibility rather than as evidence of clinical efficacy [44,45].

#### 4.5.2. Arils and Juice

Arils and juices derived primarily from aril tissue are distinguished by their high anthocyanin content, which underpins both the characteristic coloration of pomegranate products and a substantial proportion of their antioxidant capacity, alongside contributions from other classes of polyphenols [46,47]. From a translational perspective, juice represents the most common form of pomegranate consumption evaluated in human studies, lending particular relevance to its consideration in preventive health contexts. At the same time, the chemical profile of products labeled as “pomegranate juice” is highly sensitive to processing practices. Juices produced with partial inclusion of peel or membranous tissues may contain markedly higher levels of ellagitannins than those obtained exclusively from arils, which complicates direct comparison of outcomes across studies [47,48]. Critical evaluations of human intervention trials further indicate that reported clinical effects are heterogeneous across endpoints and frequently constrained by incomplete product characterization, as well as by inter-individual differences in polyphenol metabolism. In particular, variability in gut microbiota composition and the resulting capacity to generate urolithins appear to modulate systemic responsiveness to pomegranate polyphenols. Collectively, these observations support the view that “pomegranate” should be approached as a set of chemically and biologically distinct formulations rather than as a uniform dietary intervention [39,49].

#### 4.5.3. Seeds and Seed Oil

Pomegranate seeds, and especially the oil extracted from them, are characterized by a lipid-phase phytochemical profile dominated by conjugated linolenic acids, with punicic acid representing the principal component. This compositional distinction is pharmacologically relevant, as lipid-soluble constituents from seed oil display absorption patterns and tissue distribution profiles that differ substantially from those of the high-molecular-weight tannins enriched in peel-derived preparations [35]. Within the literature, punicic acid has been repeatedly discussed as a mechanistically plausible mediator of effects related to inflammation and metabolic regulation, providing a rationale for the frequent positioning of pomegranate seed oil within metabolic and inflammatory health frameworks rather than in antimicrobial applications. As emphasized in broader reviews, however, the translational relevance of seed-oil products remains contingent on factors such as formulation quality, dosing strategies, and rigorous chemical standardization, which are critical for reproducibility and meaningful comparison across studies [36,50].

#### 4.5.4. Bark, Flowers and Other Non-Fruit Tissues

In contrast to peel, arils, and seed oil, non-fruit tissues of pomegranate—such as bark and flowers—have received comparatively limited and less standardized investigation. Existing studies nonetheless suggest that these materials may contain phenolic fractions and other constituents with antioxidant and antimicrobial potential. Interpretation of these findings is complicated by substantial variability in plant material, extraction approaches, and analytical methods, which restricts direct comparison across studies [14,51]. Consequently, bark- and flower-derived preparations are best regarded as promising yet relatively under-validated sources of bioactivity when compared with the more extensively characterized peel-ellagitannin and seed-oil axes.

**Table 1:** Structured cross-matrix summary of the major biologically active constituents of *P. granatum* L., with representative quantitative ranges from primary studies, typical biological functions, and indicative evidence level. Units were harmonized where feasible; where direct harmonization was not possible because of methodological heterogeneity, values are reported as in the original studies.

Bioactive Constituent/Class	Arils/Juice	Peel	Seeds/Seed Oil	Bark/Flowers	Main Biological Functions (Typical)	Evidence Level (Typical)	Key References
Ellagitannins (punicalagin/punicalin; hydrolyzable tannins)	Punicalagin in commercial juices (processing-dependent): 0.007–0.3 g/L (6 brands); previously reported 0.017–2 g/L	Punicalagin-α: 128.6–154.9 mg/g; punicalagin-β: 200.2–216.4 mg/g (peel, DW) Across 9 cultivars: punicalagin 28.0–104.1 mg/g	Low/trace: punicalagin-α 1.15–2.93 mg/g; punicalagin-β 1.10–1.75 mg/g (seeds, DW) Seed oil: N/A	Limited quantitative datasets; phenolic/tannin-rich profiles reported for male flowers (extracts)	Antioxidant; anti-inflammatory; antimicrobial	Mostly in vitro/in vivo; human evidence indirect (food matrices, not purified ETs)	[20,21,40,42,43,44,45,52,53,54]
Ellagic acid (free and glycosides)	Ellagic acid in juices (free form; no hydrolysis): 23.43–95.02 mg/L	32.14–35.00 mg/g (peel, DW)	1.17–1.60 mg/g (seeds, DW)	Reported in non-fruit tissues (variable; limited quantification)	Antioxidant; anti-inflammatory; photoprotective (preclinical)	Mostly in vitro/in vivo; human relevance depends on bioavailability/metabolism	[14,20,40,41,52,55]
Urolithins (A/B; microbiota-derived metabolites)	N/A (host-derived metabolites after ET intake)	N/A	N/A	N/A	Anti-inflammatory; mitophagy/healthy-aging pathways; neuroprotective hypotheses	Human PK/metabolomics evidence; strong inter-individual variability (microbiota-dependent)	[29,39,49,50,56]
Anthocyanins (e.g., cyanidin/delphinidin glycosides)	Total anthocyanins (TA): 56.6–188.7 mg/L (18 juices) Example juice: total monomeric anthocyanins 42.0 mg/L (Cy3G eq.)	Detected in peel extracts (typically << juice; cultivar and extraction dependent)	N/A	N/A	Antioxidant; vascular/cardiometabolic hypotheses	Human evidence mixed; many endpoints remain preclinical or formulation-dependent	[14,46,47,48,55,57]
Flavonols (quercetin, kaempferol derivatives)	Quercetin: 0.60–5.61 mg/L; rutin: 0.68–3.12 mg/L (juice) Aril extract (5 genotypes): quercetin 6.47–27.66 μg/mL	Peel extracts (mg/g DW): quercetin 0.04; kaempferol 0.02; rutin 1.23–2.41; hyperoside 3.76–5.58	N/A (typically low)	Variable; reported in flower extracts (profile studies)	Anti-inflammatory; antioxidant; antiproliferative (preclinical)	Mostly in vitro/in vivo (extracts); limited dose–response translation to humans	[40,54,55,57,58]
Phenolic acids (gallic, caffeic, chlorogenic, etc.)	Free forms in juice (mg/L): gallic 12.42–88.51; chlorogenic 0.90–2.63; caffeic 0.16–1.64; ferulic 0.41–4.78	Gallic acid: 1.86–2.23 mg/g (peel, DW) Peel extracts (mg/g DW): gallic 0.79–4.21; ferulic 0.93–1.17; p-coumaric 0.04–0.19	Gallic acid: 0.13–0.20 mg/g (seeds, DW); seed oil: trace phenolics	Variable; under-standardized	Antioxidant; additive redox effects; antimicrobial contribution (context-dependent)	Mostly in vitro/in vivo; composition strongly affected by cultivar and processing	[20,40,41,48,52,55,58]
Seed-oil fatty acids (punicic acid; conjugated linolenic acid)	N/A	N/A	Cold-pressed seed oil (% wt): punicic acid 73.19 ± 0.50; linoleic 7.36 ± 0.16; oleic 7.69 ± 0.13; palmitic 3.92 ± 0.09; stearic 3.00 ± 0.07 Literature: punicic acid 72.4–84.1 wt% (major FA)	N/A	Metabolic modulation; anti-inflammatory hypotheses	In vivo and limited human data; effects sensitive to dose/formulation and background diet	[35,36,59]
Other phenolic reservoirs in non-fruit tissues (bark/flowers)	N/A	Minor/non-dominant compared with peel; depends on tissue definition and extraction	N/A	Bark/flowers: phenolic-rich extracts reported; bioactivity studies exist but standardization is limited	Antioxidant; antimicrobial/anti-inflammatory (context-dependent)	Under-standardized; mostly in vitro (variable extract composition)	[14,29,47,50,51,54]

N/A: not a plant constituent (produced post-ingestion).

#### 4.5.5. Phenolic Acids as Cross-Cutting Contributors and the Role of Extraction and Processing

Lower-molecular-weight phenolic acids, including gallic and caffeic acids, are detected across multiple pomegranate matrices, although they are often more prominent in peel-associated profiles. These compounds can contribute additively to redox-related effects and may modulate overall antioxidant outcomes depending on their relative abundance within a given preparation [48]. Importantly, experimentally measured “antioxidant activity” should not be interpreted as a fixed or intrinsic attribute of the plant itself. Rather, it represents an emergent, formulation-dependent phenotype shaped by the balance between tannins, pigments, and phenolic acids, as well as by processing parameters such as the pressing method, solvent composition, and extraction conditions [20,41,47].

Overall, the literature shows that pomegranate-based interventions are not directly comparable, because different studies use different formulations, doses, and study populations, which can lead to different biological effects. Preparations derived from different plant fractions are not interchangeable: peel extracts are typically ellagitannin-rich, aril-derived juices are relatively enriched in anthocyanins, peel-inclusive juices contain higher hydrolyzable tannin loads, whereas seed oil is dominated by punicic acid. These compositional differences are further amplified by extraction solvent, processing conditions, phytochemical standardization, daily dose, and intervention duration. In human studies, outcome variability is additionally shaped by population-related factors, including baseline metabolic and inflammatory status, underlying disease context, background diet, and interindividual variability in gut microbiota-dependent ellagitannin metabolism to urolithins. Accordingly, discrepancies across studies should be interpreted as formulation- and population-dependent rather than as evidence for a uniform effect of pomegranate as a whole.

## 5. Biological Properties of *P. granatum* Extracts

**Figure 1:**
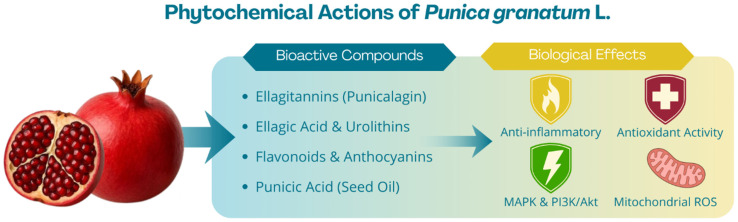
Schematic overview of the major anatomical fractions of *P. granatum* L. and their dominant phytochemical groups, including ellagitannins in the peel, anthocyanins and flavonols in arils/juice, and punicic acid-rich lipids in seed oil, together with the principal biological activities attributed to these constituents, such as antioxidant, anti-inflammatory, antimicrobial, metabolic, anti-aging, and anticancer effects.

Interest in *P. granatum* stems from its exceptionally rich phytochemical profile, particularly its high content of polyphenolic compounds that exhibit strong biological activity [60]. All parts of the pomegranate plant—including fruit juice, peel, seeds, bark, and roots—have been used for medicinal purposes, with each part offering distinct bioactive constituents and therapeutic applications [11,61,62]. Figure 1 provides an integrated overview of the phytochemical diversity of *P. granatum* and its multi-target biological activity. The scheme links the main anatomical fractions of the fruit, including peel, arils/juice, and seeds/seed oil, with their dominant groups of bioactive compounds, such as ellagitannins, anthocyanins, flavonoids, phenolic acids, and conjugated fatty acids. It also summarizes the principal biological effects associated with these fractions, including antioxidant, anti-inflammatory, antimicrobial, metabolic, anti-aging, and anticancer activities. The figure is intended to emphasize that the health-promoting potential of pomegranate depends on tissue-specific composition and on the combined action of multiple phytochemicals rather than on a single compound alone.

### 5.1. Traditional Medicinal Uses

Traditional medical systems across Eurasia have employed *Punica granatum* for centuries in a wide range of therapeutic applications. In Persian–Islamic medicine, the plant was used as a tonic to strengthen gastric function and to treat gastrointestinal disorders, respiratory diseases, skin wounds, reproductive health problems, and metabolic disturbances [62]. Similar applications are reported in Chinese and Mexican traditions, where the pomegranate exocarp has been used in the treatment of diarrhea and dysentery, which is associated with the presence of bioactive compounds such as punicalagin [11,62]. In Uyghur and Tibetan medicine, as well as in broader Chinese folk medicine, preparations derived from pomegranate seeds, peels, and flowers are used to treat conditions described as “stomach cold,” inflammatory states, and pain. This knowledge is reflected in Indian Ayurveda and Unani systems, where *P. granatum* is primarily used in the management of gastrointestinal disorders and in wound healing processes. Other ethnic traditions, including Mongolian and Hmong medicine, attribute antidiarrheal and anti-inflammatory effects on the gastric mucosa and hemostatic properties to pomegranate [11,62]. Across diverse traditional medical systems, pomegranate is consistently characterized as an astringent, drying, and cooling agent applied in the treatment of disorders of the digestive, respiratory, dermatological, reproductive, and metabolic systems. Many traditional formulations containing *P. granatum* remain in use today [11,62]. The extensive and long-standing use of this plant across cultures underscores its recognized therapeutic value and provides important rationale for modern scientific investigation.

### 5.2. Antibacterial Properties

Pomegranate peel extract has been increasingly investigated for its antibacterial activity, with numerous studies demonstrating high efficacy, particularly against Gram-positive pathogens. The literature identifies punicalagin as one of the key compounds responsible for the antibacterial activity of pomegranate peel extracts [41]. This compound exhibits low minimum inhibitory concentration (MIC) values (16 µg/mL) against multiple strains of *Staphylococcus aureus* [42]. Punicalagin effectively disrupts bacterial cell membrane integrity and induces iron-deprivation stress, as evidenced by increased expression of genes involved in Fe^2+^ acquisition [63]. Punicalagin also demonstrates notable activity against antibiotic-resistant bacterial pathogens. Studies have revealed a multitarget mode of action against methicillin-resistant *S. aureus* (MRSA). Specifically, punicalagin destabilizes the bacterial cell wall through modulation of genes involved in peptidoglycan synthesis and lipoteichoic acid biosynthesis, disrupts membrane function, impairs lipid biosynthesis, and downregulates the expression of numerous ribosomal genes, thereby limiting protein translation [63]. Antibacterial activity of pomegranate peel extracts has also been observed against other Gram-positive bacteria. In assays involving *Bacillus subtilis*, *Bacillus cereus*, and *Enterococcus faecalis*, extracts not only inhibited biofilm formation but also eradicated pre-formed biofilms. Acidified methanolic and ethanolic extracts exhibited the strongest effects, reducing biofilm formation by up to 80% [64]. Aqueous pomegranate peel extracts also showed antibacterial activity against *Listeria monocytogenes*, with MIC values of 19 mg/mL for the aqueous extract and 24 mg/mL for the ethanolic extract, which was attributed to the higher phenolic content of the aqueous preparation [65]. The literature further emphasizes the broad-spectrum antimicrobial activity of pomegranate peel extracts against Gram-negative bacteria. In one study, methanolic peel extracts exhibited MIC values ranging from 3.9 to 7.8 mg/mL against members of the family *Enterobacteriaceae*, including *Escherichia coli*. Moreover, punicalagin used in combination with conventional antibiotics (aminoglycosides, β-lactams, and fluoroquinolones) significantly enhanced their antibacterial efficacy [24]. In Gram-negative bacteria, punicalagin also exerts membrane-targeting effects. It induces loss of membrane integrity, significant membrane depolarization, and disruption of intracellular pH homeostasis, leading to destabilization of metabolic processes in *Salmonella typhimurium* [66]. Studies also confirm the antimicrobial potential of pomegranate peel extracts against *Pseudomonas aeruginosa*. In this case, phenolic compounds such as punicalagin and punicalin were shown to attenuate virulence mechanisms and reduce bacterial adhesion and biofilm-forming capacity [67]. Collectively, numerous studies demonstrate that pomegranate peel extracts exhibit antibacterial activity against a wide range of bacterial species, including both Gram-positive and Gram-negative organisms. Gram-positive bacteria are generally more susceptible, as indicated by lower MIC values and stronger membrane-disrupting effects. In contrast, higher concentrations are typically required to inhibit Gram-negative bacteria, primarily due to the presence of an outer lipopolysaccharide membrane that limits the penetration of phenolic compounds and ellagitannins [42,68]. Taken together, these findings provide strong in vitro and mechanistic support for the antibacterial potential of pomegranate peel-derived compounds; however, their clinical relevance remains to be confirmed in appropriately designed in vivo and human studies.

### 5.3. Beneficial Effects on the Microbiome

**Figure 2:**
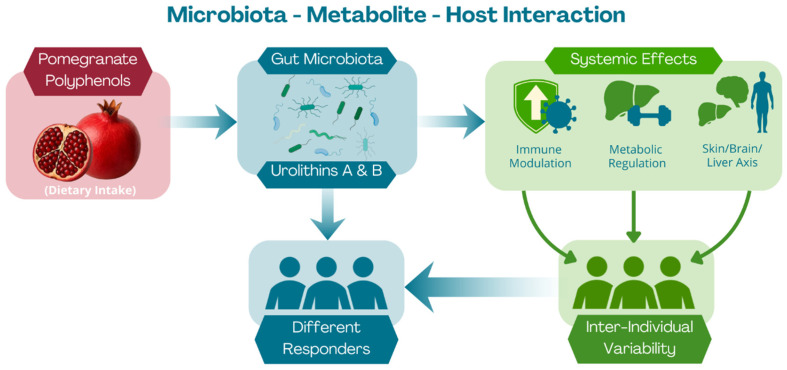
Schematic representation of the microbiota–metabolite–host axis involved in the biological effects of pomegranate polyphenols. The figure illustrates the transformation of ellagitannins and ellagic acid into urolithins by the gut microbiota, the selective modulation of microbial composition and activity, and the potential downstream effects on oxidative stress, inflammation, and metabolic homeostasis.

The gut microbiota is increasingly recognized as a key mediator of the health-promoting effects of dietary polyphenols, acting both as a biological target and a metabolic partner in shaping host redox balance, inflammation, and metabolic homeostasis. Figure 2 summarizes the microbiota-metabolite-host axis that underlies an important part of pomegranate bioactivity. In this concept, ellagitannins and ellagic acid derived from pomegranate are transformed by the gut microbiota into bioactive metabolites, particularly urolithins, which may contribute to systemic effects beyond the gastrointestinal tract. The scheme also illustrates that these interactions are bidirectional and context-dependent, involving selective modulation of microbial taxa, changes in microbial metabolite production, and downstream effects on inflammation, oxidative balance, and metabolic homeostasis. Importantly, the figure highlights that interindividual variability in gut microbiota composition may influence the magnitude and nature of the biological response to pomegranate intake. Consistently, short-term supplementation with a commercial pomegranate extract in an in vitro colon model increased overall bacterial diversity and selectively modulated specific taxa, including *Prevotella*, *Enterocloster*, and *Mitsuokella*, with minimal effects on global short-chain fatty acid production [69]. In line with these findings, pomegranate extract has been shown to exert strain-specific effects on probiotic bacteria, supporting its potential role as a functional prebiotic ingredient. In vitro studies demonstrated that pomegranate polyphenols differentially modulate growth, viability, transcriptional activity, and metabolite utilization of *Lactobacillus*, *Lacticaseibacillus*, and *Lactiplantibacillus* strains, with particularly pronounced enhancement of survival and transcriptional responsiveness in *Lactobacillus acidophilus*. Moreover, exposure to pomegranate extract induced strain-dependent utilization of punicalagin, ellagic acid, and gallic acid, highlighting selective microbial adaptation to pomegranate-derived polyphenols [70]. Furthermore, in vitro digestion and colonic fermentation of a standardized pomegranate extract markedly enhanced total phenolic content, reflecting extensive microbial biotransformation of ellagic acid-derived compounds, including urolithins. Despite a reduction in intrinsic antioxidant capacity after digestion, fermented pomegranate extract significantly increased phenolic availability and modulated gut microbiota functionality, as evidenced by elevated production of short-chain fatty acids, particularly lactic acid. Notably, phenolic enrichment and metabolite generation differed according to the microbial source, underscoring substantial interindividual variability in microbiota-driven polyphenol metabolism [71]. Evidence from animal models further supports a functional interaction between pomegranate polyphenols, gut microbiota, and host metabolism. In a high-fat diet-induced obesity model, pomegranate peel extract—rich in ellagitannins—favorably modulated gut microbiota composition and metabolic outcomes. Supplementation with both native and microencapsulated pomegranate peel extract increased gut microbial alpha diversity, enhanced systemic antioxidant capacity, and was associated with higher energy expenditure and reduced hepatic arachidonic acid content compared with a high-fat diet alone [72]. Similarly, pomegranate peel polyphenols—alone or combined with inulin—exerted pronounced anti-obesity effects by reducing body weight and hepatic and serum lipid levels. These effects were accompanied by restoration of gut microbiota balance, characterized by enrichment of short-chain fatty acid-producing taxa, including *Lactobacillus*, *Roseburia*, *Ruminococcaceae*, and *Bacteroides*, and depletion of obesity-associated bacteria. Moreover, pomegranate peel polyphenols modulated host metabolic pathways related to amino acid and arachidonic acid metabolism and attenuated high-fat diet-induced increases in triglycerides and pro-inflammatory cytokines (IL-6, TNF-α), highlighting a microbiota-metabolite-inflammation axis underlying their anti-obesity activity [73]. In addition to metabolic regulation, pomegranate-derived polyphenols have been shown to enhance microbiota resilience against pathogenic challenges. In a murine model of *Citrobacter rodentium* infection, supplementation with pomegranate peel extract altered gut microbiota composition and reduced pathogen expansion. Pomegranate treatment shifted the microbial profile toward a lower Firmicutes/Bacteroidetes ratio, decreased *Lactobacillus* abundance, and increased *Bacteroidetes*, *Proteobacteria*, and *Verrucomicrobia*, resulting in markedly reduced intestinal colonization by *C. rodentium* compared with controls [74]. Human intervention studies further corroborate the microbiota-modulating effects of pomegranate. In a dietary intervention study, three-week consumption of peeled pomegranate arils increased the relative abundance of genera associated with carbohydrate fermentation, including *Saccharofermentans*, *Enterococcus*, and *Prevotella*, while reducing *Dysosmobacter*, *Coprococcus*, and *Collinsella*. Importantly, the magnitude of microbial community shifts correlated with urinary polyphenol levels, indicating a close interplay between polyphenol bioavailability and microbial responsiveness and resulting in an improved ratio of presumed beneficial-to-detrimental taxa [75]. Moreover, evidence from a randomized controlled trial in overweight and obese individuals demonstrated that pomegranate juice combined with inulin exerted a more pronounced modulatory effect on gut microbiota composition than pomegranate juice alone, promoting the production of microbiota-derived short-chain fatty acids and pomegranate polyphenol metabolites, despite the absence of short-term changes in anthropometric or metabolic parameters [76]. Similarly, supplementation with a standardized punicalagin-rich pomegranate extract selectively modulated gut microbiota composition and microbial metabolite production in healthy adults. Although overall microbial diversity remained unchanged after four weeks, pomegranate intake increased the relative abundance of short-chain fatty acid-producing taxa, including *Faecalibacterium prausnitzii*, *Roseburia* spp., *Ruminococcus* spp., and *Coprococcus eutectus*. These microbial shifts were associated with elevated circulating propionate levels and increased production of microbiota-derived urolithins, indicating enhanced ellagitannin biotransformation [77].

Finally, emerging evidence suggests that microbiota-mediated effects of pomegranate may extend beyond the gut. In a randomized controlled trial, oral supplementation with pomegranate extract or juice for 12 weeks enhanced resistance to UVB-induced skin damage, as reflected by a significant increase in minimal erythema dose compared with placebo. Although no major shifts were observed at the phylum level, pomegranate intake induced significant changes in skin microbiota composition at the family and genus levels, including taxa potentially involved in UV absorption, such as members of the *Methylobacteriaceae* family. While a direct correlation between microbial changes and photoprotection could not be established, these findings support a potential gut-skin axis-related mechanism of action [78].

Together, these findings indicate that pomegranate-derived polyphenols modulate microbial ecology in a selective and context-dependent manner, influencing gut and extraintestinal microbiota composition and metabolic output, and potentially contributing to their systemic health benefits.

### 5.4. Antifungal Properties

The antifungal activity of pomegranate peel extracts has been widely described in the literature and confirmed by numerous studies against yeasts of the genus *Candida* as well as molds belonging to the genus *Aspergillus*. These pathogens are of particular relevance in clinical practice and food safety assessment [79]. In a study by Colombari et al., pomegranate peel extract significantly inhibited the growth of *Candida albicans*, suppressed biofilm formation, and disrupted cell-to-cell communication, confirming its antifungal potential [79]. Punicalagin and punicalin present in the extracts also exhibited antifungal activity, with MIC values of 125 µg/mL for *C. albicans* and *C. parapsilosis*, and 62.5 µg/mL for *C. tropicalis*, *C. glabrata*, and *C. krusei* [80].

In in vitro studies, punicalagin inhibited the activity of topoisomerase I and II in *C. albicans*, with half maximal inhibitory concentration (IC_50_) values of 9.0 and 4.6 µM, respectively, resulting in suppressed yeast growth. These findings suggest a multitarget mode of action of punicalagin, encompassing both disruption of cell membrane integrity and inhibition of biofilm formation, as well as interference with DNA replication mechanisms. This activity was confirmed for both reference and clinical strains [43].

Crude pomegranate peel extract, containing punicalagin, punicalin, and phenolic acids, demonstrated significant activity against *Candida* species (including *C. albicans*, *C. parapsilosis*, *C. krusei*, and *C. glabrata*), with an MIC value of 10 mg/mL. Notably, the extract also strongly inhibited biofilm formation by these fungi. Importantly, the antifungal effect was not associated with direct damage to the cell wall, suggesting that it results from the combined action of multiple polyphenolic constituents [81].

Pomegranate peel extract also showed inhibitory activity against phytopathogenic fungi responsible for fruit decay, including *Penicillium expansum*, *Botrytis cinerea*, *Aspergillus niger*, *Colletotrichum gloeosporioides*, *Monilinia fructicola*, and *Rhizopus stolonifer*. The strongest antifungal activity was observed for the n-hexane fraction of the ethanolic extract [82].

### 5.5. Antiviral Activity

Bioactive compounds present in pomegranate peel extracts also exhibit antiviral activity, particularly against influenza A virus. This activity has been demonstrated by significant inhibition of viral replication, reduction in viral RNA levels, and decreased expression of viral proteins. The mechanism of action appears to involve early stages of infection, likely through interference with viral adsorption or internalization into host cells, as well as inhibition of initial replication steps. High in vitro efficacy is reflected by IC_50_ values of 5–6 µg/mL [83].

Punicalagin has also been reported to possess antiviral activity against coronaviruses, which is attributed to its ability to interfere with viral replication proteins. Specifically, punicalagin has been shown to bind to the NSP13 helicase, an enzyme essential for RNA unwinding, thereby inhibiting its activity [84]. In addition, pomegranate peel extracts have demonstrated antiviral activity against SARS-CoV-2. In vitro studies confirmed that the extract significantly reduced binding of the viral Spike protein to the ACE2 receptor and inhibited the activity of the viral 3CL protease, indicating a dual mechanism that blocks both viral entry and replication [45]. Polyphenols present in the extract, particularly punicalagin and punicalin, also reduced viral infectivity, supporting their role as key active constituents [44].

Furthermore, studies suggest that beyond direct inhibition of viral functions, pomegranate peel extracts may modulate host responses by attenuating inflammation and oxidative stress, thereby contributing to their overall antiviral potential [85].

### 5.6. Antioxidant and Anti-Aging Properties

Oxidative stress is defined as an imbalance between the production of ROS and the ability of the organism to neutralize them. Importantly, low and tightly regulated levels of ROS, which are generated as by-products of mitochondrial respiration, serve essential physiological signaling functions, whereas excessive ROS production disrupts redox homeostasis and leads to oxidative stress-mediated damage to DNA, proteins, and lipids [86,87]. Pomegranate is recognized as one of the richest natural sources of phenolic compounds. It contains high levels of polyphenols, including punicalagin, ellagic acid, ellagitannins, flavonoids, and anthocyanins, which play a key role in the neutralization of ROS. These antioxidants significantly reduce oxidative stress, thereby protecting cells from oxidative damage [88]. High concentrations of ellagitannins have been identified in the pomegranate peel, ranging from 193 to 420 mg/g of dry weight, along with flavonoid levels of 84–134 mg/g, making the pericarp the most antioxidant-rich part of the fruit. The bioactive polyphenols of pomegranate exert their effects by stabilizing free radicals, inhibiting lipid peroxidation, and limiting the formation of oxidative damage to DNA, proteins, and cellular membranes, collectively constituting a protective barrier against aging processes and the detrimental effects of oxidative stress [89].

Pomegranate fruit extracts, including those derived from the peel and seeds, exhibit significant antioxidant potential. This activity is reflected by a reduction in malondialdehyde (MDA) levels—a key marker of lipid peroxidation—as well as by the stimulation of enzymatic antioxidant defense systems, such as superoxide dismutase (SOD), catalase (CAT), and glutathione peroxidase (GPx) [90]. In experimental models, oral supplementation with pomegranate seed juice exerted significant protective effects in streptozotocin-nicotinamide-induced diabetic rats by restoring antioxidant enzyme activities (SOD, CAT), reducing oxidative stress markers (MDA), improving glycemic and insulin status, and ameliorating histopathological alterations, indicating a strong antioxidative potential against diabetes-associated oxidative stress [91]. Similarly, in streptozotocin-induced diabetic rats, dietary pomegranate supplementation exerted a pronounced renoprotective effect by reducing oxidative stress and lipid peroxidation, normalizing renal function markers and glycemia, upregulating antioxidant defense genes (SOD, CAT, glutathione reductase, GPx), and downregulating NADPH oxidase subunits and NF-κB signaling, ultimately ameliorating diabetes-associated kidney injury [92].

Beyond diabetes-related models, pomegranate-derived preparations have demonstrated protective effects in other metabolic and organ-specific contexts. In a high-fat-high-fructose diet-induced obesity model, pomegranate seed oil, leaves, juice, and peel extracts exerted neuroprotective effects by inhibiting brain cholinesterase activity and attenuating oxidative stress through reduced MDA and protein carbonylation levels alongside enhanced SOD and GPx activity, concomitantly improving lipid profile and preventing lipid accumulation in the brain [93]. Likewise, polyphenol-rich pomegranate peel extract modulated redox balance and immune-related signaling in the spleen of rats with metabolic syndrome by reducing ROS/reactive nitrogen species and MDA levels, enhancing catalase and glutathione peroxidase activity, regulating inflammatory mediators (IL-6, NF-κB, TNFα), and altering the BAX/BCL-2 apoptotic balance, indicating its potential to counteract oxidative stress–driven immune dysfunction associated with metabolic syndrome [94]. Moreover, pomegranate juice supplementation markedly attenuated ischemia–reperfusion-induced oxidative stress in a rat model of testicular torsion–detorsion by reducing serum and testicular MDA and SOD levels while simultaneously improving spermatogenic cell counts, indicating a protective effect on testicular redox balance and sperm quality [95]. Cardiovascular protection has also been reported. In a spontaneously hypertensive rat model, pomegranate peel extract exerted antihypertensive and vasculoprotective effects by reducing systolic blood pressure and coronary angiotensin-converting enzyme activity, lowering superoxide anion levels, and attenuating vascular remodeling, thereby protecting against hypertension- and aging-associated oxidative stress and coronary damage [96].

The attenuation of oxidative stress has further been demonstrated in the context of physical exercise and aging. Supplementation with pomegranate juice effectively limited the exercise-induced increase in MDA levels as well as the acute intensification of lipid peroxidation following resistance exercise [97]. Evidence from randomized controlled clinical trials indicates that pomegranate peel supplementation enhances post-exercise recovery by reducing oxidative stress through lowering MDA levels and increasing antioxidant enzyme activity [98]. In addition, pomegranate extract supplementation attenuates age-related oxidative stress by reducing both cytoplasmic and mitochondrial ROS levels in leukocytes of aged mice, counteracting oxidant-induced hydrogen peroxide accumulation and protecting neutrophils, thereby potentially supporting immune function and promoting healthy aging [99]. Furthermore, pomegranate juice extract mitigated 5-fluorouracil-induced oxidative stress and inflammation in human keratinocytes by suppressing ROS and nitrotyrosine formation, activating cytoprotective antioxidant enzymes (heme oxygenase, NAD(P)H:quinone oxidoreductase 1), and inhibiting NF-κB signaling and pro-inflammatory cytokine release while reducing apoptosis and promoting wound repair, supporting its potential as an adjuvant against chemotherapy-induced skin toxicity [100]. The evidence summarized above derives predominantly from in vitro systems and animal models and should therefore be interpreted primarily as mechanistic and preclinical support rather than as direct proof of clinical efficacy.

Clinical evidence further supports, but also nuances, these findings. In a randomized clinical trial involving 60 patients with type 2 diabetes, six weeks of daily pomegranate juice consumption significantly reduced oxidized LDL and anti-oxidized LDL antibodies while increasing total antioxidant capacity and paraoxonase arylesterase activity, indicating a favorable modulation of oxidative stress [101]. In line with these results, a meta-analysis of 33 randomized controlled trials demonstrated that pomegranate consumption significantly reduces key inflammatory biomarkers (CRP, IL-6, TNF-α) and the lipid peroxidation marker MDA, while modestly increasing total antioxidant capacity, supporting its beneficial role in attenuating oxidative stress and inflammation, particularly in adults with cardiovascular diseases [102]. Moreover, pomegranate juice consumption exerted potent antiatherogenic effects in both healthy humans and apolipoprotein E-deficient mice by reducing LDL oxidation, aggregation, and macrophage uptake, enhancing paraoxonase activity, limiting foam cell formation, and markedly decreasing atherosclerotic lesion size [103].

Nevertheless, the overall clinical evidence remains heterogeneous. A separate meta-analysis of 11 randomized controlled trials involving 484 participants did not provide convincing evidence that pomegranate intake significantly improves systemic antioxidant status, as no statistically significant effects were observed for total antioxidant capacity, GPx, paraoxonase activity, or MDA levels, despite trends toward antioxidant benefit [104]. Consistently, a systematic review reported that although pomegranate supplementation tended to reduce oxidative stress biomarkers and significantly increased GPx activity and total antioxidant capacity, effects on oxidized LDL and paraoxonase-1 were not statistically significant, and the overall quality of evidence was low due to substantial heterogeneity in formulation, dosage, intervention duration, and study populations, predominantly from Eastern countries [105]. In addition, a randomized controlled trial in patients with type 2 diabetes mellitus showed that 12-week consumption of bread fortified with pomegranate peel powder did not significantly improve oxidative stress markers or inflammatory status after adjustment for confounders, despite modest within-group reductions in hs-CRP and depressive symptoms, underscoring the context-dependent and formulation-specific nature of the clinical effects of pomegranate-derived products [106]. Taken together, these discrepancies should not be interpreted as simple inconsistency in the pomegranate literature but rather as a predictable consequence of substantial heterogeneity across clinical studies. Differences in formulation type (e.g., juice, extract, peel-fortified foods, or seed oil), phytochemical standardization, daily dose, intervention duration, and endpoint selection substantially influence the observed response. Moreover, participant-related factors, including baseline oxidative and inflammatory status, metabolic disease burden, dietary background, medication use, and interindividual variability in gut microbiota-mediated ellagitannin metabolism to urolithins, may determine whether a measurable benefit is detected. Therefore, the available clinical evidence should be interpreted in a formulation- and population-dependent manner rather than as support for a uniform antioxidant effect of all pomegranate-derived products.

Mechanistic studies suggest that the mechanism of action of pomegranate extracts may involve two complementary and interrelated pathways. The first is the direct scavenging of ROS by abundant polyphenols, which limits lipid peroxidation and oxidative macromolecular damage. The second, and potentially more relevant from a regulatory perspective, involves activation of the Nrf2 signaling pathway. Nrf2 translocation to the nucleus induces the expression of antioxidant and cytoprotective genes encoding phase II detoxifying enzymes and redox-regulating proteins, including superoxide dismutase, catalase, glutathione peroxidase, and heme oxygenase-1. Importantly, Nrf2 activation is tightly linked to suppression of NF-κB–mediated inflammatory signaling, highlighting the role of pomegranate polyphenols as adaptive redox modulators rather than simple radical scavengers [97,107,108]. Moreover, chronic pomegranate extract supplementation alleviates hypertension by reducing oxidative stress and inflammation in the paraventricular nucleus through AMPK-Nrf2 pathway activation, leading to decreased mitochondrial superoxide production, improved mitochondrial function and dynamics, and consequent reductions in blood pressure and cardiac hypertrophy [109].

Importantly, not all mechanisms attributed to pomegranate-derived products are supported to the same evidentiary degree. In humans, the best-supported mechanistic aspect is the microbiota-mediated conversion of ellagitannins and ellagic acid into urolithins, which are likely to account for an important part of systemic bioactivity. By contrast, direct ROS scavenging and the modulation of signaling pathways such as Nrf2, NF-κB, AMPK, mTOR, and apoptosis-related cascades are supported mainly by in vitro and animal studies and should therefore be interpreted as mechanistically plausible rather than clinically established. Their translational relevance is further constrained by limited bioavailability of intact high-molecular-weight polyphenols and by strong dependence on matrix, processing, formulation, and host metabolic capacity. In addition, inter-individual differences in gut microbiota composition and urolithin-producing metabotypes may substantially contribute to the heterogeneous responses observed across human studies.

Preclinical studies suggest that pomegranate extracts modulate the expression of genes associated with inflammation and oxidative stress—key processes underlying aging and longevity. These compounds may influence the activity of enzymes and receptors involved in these pathways, thereby supporting cellular repair mechanisms and extending cellular lifespan [110]. As discussed earlier, the strong antioxidant properties of pomegranate polyphenols—such as punicalagin and ellagic acid—significantly reduce oxidative stress, a major driver of aging. These compounds decrease levels of pro-inflammatory cytokines that increase during aging and chronic disease and enhance skin health. Limited clinical studies suggest that pomegranate extracts improve skin hydration, elasticity, and overall condition, reducing visible signs of aging [50,111].

A key component of the anti-aging activity of pomegranate is its protective effect against photoaging induced by UVA and UVB radiation. Pomegranate peel extracts and ellagic acid have been shown to reduce markers of DNA damage, such as cyclobutane pyrimidine dimers (CPDs) and 8-hydroxy-2′-deoxyguanosine (8-OHdG), while simultaneously inhibiting lipid peroxidation and preventing UVB-induced depletion of glutathione levels in the skin. Moreover, pomegranate significantly influences the activity of signaling proteins associated with cellular stress and proliferation, including mTOR, thereby limiting adverse structural and biochemical changes associated with UVA exposure [112]. Pomegranate also exerts beneficial effects on key genes involved in aging. Studies have shown that in aged animals, the expression of *TERT* and *Txnrd1* genes is reduced, contributing to telomere shortening and impaired antioxidant defense. Supplementation with pomegranate peel extract significantly increased the expression levels of both genes, supporting telomere maintenance and redox regeneration and thereby slowing aging processes [113]. Ellagic acid has been shown to exhibit significant potential in increasing collagen content and reducing the expression of matrix metalloproteinase-1 (MMP-1), an enzyme responsible for collagen degradation [114]. In addition, urolithins derived from ellagitannins present in pomegranate peel and juice counteract neurodegenerative processes and support cognitive health during aging [50]. In summary, the collective preclinical and clinical evidence indicates that pomegranate-derived polyphenols act as multifaceted redox and stress-response modulators, linking antioxidant defense, anti-inflammatory signaling, and cytoprotective gene regulation to the attenuation of oxidative damage and the promotion of healthy aging, albeit in a formulation- and context-dependent manner.

### 5.7. Anticancer Properties

A key feature of the antioxidant activity of pomegranate is its ability to exert a dual, cell-type-dependent effect. In normal cells with preserved metabolic homeostasis, pomegranate bioactive compounds act protectively. In contrast, in cancer cells they may induce oxidative stress, leading to cellular damage. The anticancer properties of pomegranate arise from two overlapping processes: pro-oxidative activity and activation of apoptotic pathways. Pomegranate extracts increase intracellular ROS levels, thereby triggering signaling cascades involving apoptotic proteins such as p53, cytochrome c, and caspase-3. Concurrently, the expression of the anti-apoptotic gene *Bcl-2* is reduced, promoting programmed cancer cell death through increased mitochondrial membrane permeability and cytochrome c release into the cytoplasm [115].

This mechanistic model is supported by in vitro studies, including those related to non-alcoholic fatty liver disease–associated hepatocellular models. It has been demonstrated that pomegranate peel and seed extracts induce strong cytotoxic effects in HepG2 hepatoma cells. This effect is mediated by enhanced oxidative stress, manifested by increased ROS and MDA levels, accompanied by decreased activity of key antioxidant enzymes (SOD, GPx, and CAT). Disruption of redox homeostasis under these conditions leads to activation of apoptotic pathways in cancer cells [90].

Additional studies have revealed the anticancer activity of pomegranate peel polyphenols in cervical cancer models, including cancers associated with human papillomavirus (HPV) infection. Polyphenols such as ellagic acid and punicalagin induce cell-cycle arrest at the G0/G1 and G1 phases, activate apoptotic pathways, and inhibit the expression of viral oncogenes E6 and E7, which are responsible for initiating cervical carcinogenesis. Pomegranate polyphenols inhibit key signaling pathways involved in cancer cell proliferation, survival, and invasion, including NF-κB, PI3K/Akt/mTOR, JAK/STAT3, and Wnt/β-catenin pathways, confirming their broad anticancer potential [116]. In studies on prostate (PC3), colorectal (HCT116), and ovarian (SKOV-3) cancer cell lines, six pomegranate peel extracts exhibited strong cytotoxic activity against these malignancies. The most potent effects were observed for aqueous and methanolic extracts, which demonstrated very low IC_50_ values, particularly against PC3 cells (0.1 µg/mL), indicating exceptional anticancer efficacy. A high-temperature aqueous extract also inhibited the proliferation of HCT116 cells (IC_50_ = 21.45 µg/mL) [117].

## 6. Conclusions and Perspective

*P. granatum* L. represents a botanically and pharmacologically relevant species distinguished by its high content of structurally diverse bioactive compounds and a broad spectrum of reported biological activities. Accumulated evidence indicates that preparations derived from different fruit tissues—particularly the peel and seeds—exert antimicrobial, antioxidant, anti-inflammatory, metabolic, anti-aging, and anticancer effects. These activities arise from a complex phytochemical matrix dominated by ellagitannins, ellagic acid derivatives, flavonoids, anthocyanins, and characteristic fatty acids, supporting a multi-component and multi-target mode of action rather than a single-compound paradigm. At the mechanistic level, modulation of redox homeostasis and inflammatory signaling emerges as a central axis of pomegranate bioactivity, with polyphenols acting both as direct antioxidants and as regulators of endogenous cytoprotective pathways. The resulting biological effects are highly context-dependent, conferring protection in normal tissues while promoting stress-associated responses in malignant cells. Increasing attention is also being directed toward interactions with the gut microbiota, which transform pomegranate polyphenols into bioactive metabolites and may partly explain interindividual variability in clinical outcomes. Despite strong mechanistic and preclinical support, the clinical translation of these findings remains limited. Future progress will require more rigorous human studies integrating pharmacokinetic, metabolite-centered, and host-related determinants of response. With such advances, selected pomegranate-derived products—particularly polyphenol-rich peel extracts and seed oil—may become better defined candidates for evidence-based nutraceutical or adjunct therapeutic use. However, the currently available human evidence remains heterogeneous, and future studies should place greater emphasis on chemically standardized formulations, marker-based dose comparability, intervention duration, and population stratification according to baseline metabolic status and microbiota-dependent metabolite production.

## Data Availability

No new data were created or analyzed in this study. Data sharing is not applicable to this article.

## Abbreviations

The following abbreviations are used in this manuscript:

ACE	Angiotensin-converting enzyme
ACE2	Angiotensin-converting enzyme 2
AMPK	AMP-activated protein kinase
BAX	BCL-2-associated X protein
BCL-2	B-cell lymphoma 2
CAT	Catalase
CRP	C-reactive protein
Cy3G eq.	Cyanidin-3-glucoside equivalents
DNA	Deoxyribonucleic acid
DW	Dry weight
ET	Ellagitannin
ETs	Ellagitannins
FA	Fatty acid
GAE	Gallic acid equivalents
GC–MS	Gas chromatography–mass spectrometry
GPx	Glutathione peroxidase
HPLC	High-performance liquid chromatography
HPV	Human papillomavirus
hs-CRP	High-sensitivity C-reactive protein
IC50	Half maximal inhibitory concentration
IL-6	Interleukin-6
JAK/STAT3	Janus kinase/signal transducer and activator of transcription 3
LDL	Low-density lipoprotein
MDA	Malondialdehyde
MIC	Minimum inhibitory concentration
MMP-1	Matrix metalloproteinase 1
MRSA	Methicillin-resistant Staphylococcus aureus
NADPH	Nicotinamide adenine dinucleotide phosphate
NF-κB	Nuclear factor kappa B
NMR	Nuclear magnetic resonance
Nrf2	Nuclear factor erythroid 2-related factor 2
PI3K/Akt/mTOR	Phosphoinositide 3-kinase/protein kinase B/mammalian target of rapamycin
PK	Pharmacokinetic
RNA	Ribonucleic acid
ROS	Reactive oxygen species
SARS-CoV-2	Severe acute respiratory syndrome coronavirus 2
SOD	Superoxide dismutase
TA	Total anthocyanins
TE	Trolox equivalents
TERT	Telomerase reverse transcriptase
TNF-α	Tumor necrosis factor alpha
Txnrd1	Thioredoxin reductase 1
UHPLC–MS	Ultra-high-performance liquid chromatography–mass spectrometry
UVA	Ultraviolet A
UVB	Ultraviolet B
Wnt/β-catenin	Wingless/Integrated-beta-catenin signaling pathway
